# A three-species synthetic community model whose rapid response to antagonism allows the study of higher-order dynamics and emergent properties in minutes

**DOI:** 10.3389/fmicb.2023.1057883

**Published:** 2023-06-02

**Authors:** Bernardo Aguilar-Salinas, Gabriela Olmedo-Álvarez

**Affiliations:** Departamento de Ingeniería Genética, Unidad Irapuato, Centro de Investigación y de Estudios Avanzados del IPN, Irapuato, Mexico

**Keywords:** higher-order interactions, bacterial antagonism, synthetic community, emergent properties, immediate response

## Abstract

Microbial communities can be considered complex adaptive systems. Understanding how these systems arise from different components and how the dynamics of microbial interactions allow for species coexistence are fundamental questions in ecology. To address these questions, we built a three-species synthetic community, called BARS (Bacillota A + S + R). Each species in this community exhibits one of three ecological roles: Antagonistic, Sensitive, or Resistant, assigned in the context of a sediment community. We show that the BARS community reproduces features of complex communities and exhibits higher-order interaction (HOI) dynamics. In paired interactions, the majority of the S species (*Sutcliffiella horikoshii* 20a) population dies within 5 min when paired with the A species (*Bacillus pumilus* 145). However, an emergent property appears upon adding the third interactor, as antagonism of species A over S is not observed in the presence of the R species (*Bacillus cereus* 111). For the paired interaction, within the first 5 min, the surviving population of the S species acquires tolerance to species A, and species A ceases antagonism. This qualitative change reflects endogenous dynamics leading to the expression for tolerance to an antagonistic substance. The stability reached in the triple interaction exhibits a nonlinear response, highly sensitive to the density of the R species. In summary, our HOI model allows the study of the assembly dynamics of a three-species community and evaluating the immediate outcome within a 30 min frame. The BARS has features of a complex system where the paired interactions do not predict the community dynamics. The model is amenable to mechanistic dissection and to modeling how the parts integrate to achieve collective properties.

## 1. Introduction

Microbial communities form the basis of all biological systems and are dynamic and complex. The complexity of microbial communities results, among other things, from the great diversity of interacting species. The stability in highly diverse communities is proposed to be the result of systems of more than two competitors forming a network of competitive relationships, the structure of which should influence the dynamics of the system as a whole (Bissett et al., 2013; Konopka et al., 2015). The stability of communities is considered an emergent property. Extensive effort has been dedicated to understanding the processes that contribute to community assembly (Reviewed in Nemergut et al., 2013). Synthetic ecology offers an approach to studying communities, reducing the number of variables, particularly the number of interacting species, the environment, and the spatial setting. One critical aspect, though often underappreciated, is that synthetic ecology allows controlling the timing of the experiments.

Competitive interactions are a defining characteristic of microbial communities, and competition can be indirect, through competition for nutrients (exploitative competition) or direct, when one individual harms another (interference competition; Hibbing et al., 2010; Cornforth and Foster, 2013). Individuals possess unique genetic repertoires that allow them to respond rapidly to environmental changes and perceived threats from other community members. For instance, they can synthesize siderophores to compete for nutrients, such as iron (Wandersman and Delepelaire, 2004). For direct competition, bacteria can produce antibiotics, toxins, and surfactants (Yim et al., 2006; Cornforth and Foster, 2013; Niehus et al., 2021). Antimicrobial compounds mediate interactions between different species, related strains of the same species, or genetically identical individuals in a population (Kerr et al., 2002; Abrudan et al., 2015; Stubbendieck and Straight, 2015; Lyons and Kolter, 2017), and some have evolved strategies for delivering antibiotics and toxins in a cell-to-cell contact fashion, such as type VI secretion (T6S) and contact-dependent growth inhibition (CDI) (Garcia, 2018). Understanding the diverse adaptation strategies of microbial communities to compete and survive is essential for comprehending the functioning and evolution of microbial ecosystems.

A common approach to studying interactions among microbes is through the analysis of pairwise interactions. This strategy was, after all, the source of discovery of natural products encoded by bacteria and fungi that have been the sources of many antibiotics in current clinical use. Pairwise interactions are commonly studied to understand microbe interactions, but modeling multispecies communities from paired interactions has limitations (Carrara et al., 2015). Friedman et al. (2017) suggest that species that coexist in pairs will survive, and those excluded by any surviving species will go extinct in a multispecies setting. However, in higher-order interactions, the interactions between a group of species depend on the presence and interactions of other species in the community. The presence of additional species can modulate interactions, making predictions from paired interactions complicated (Billick and Case, 1994).

Simplified microbial models have been widely employed to study community coexistence and assembly. One example is the non-transitive rock-paper-scissors model developed by Kerr et al. (2002) using three isogenic *E. coli* strains (toxin-producer, toxin-sensitive, and toxin-resistant strain). This model demonstrated that coexistence can occur in spatially structured environments, but not in mixed environments where microbes are continuously dispersed and interact directly and indirectly through diffusible molecules, leading to unstable coexisting communities (Kerr et al., 2002). Kerr’s model uses a single species and isogenic laboratory strains with a single trait altered, which is useful for studying specific mechanisms. Another synthetic community is the “THOR” model by Lozano et al. (2019), which explores paired interactions with different species that have an antagonistic effect on colony expansion and biofilm formation. However, Lozano’s model evaluated the antagonistic interaction only after 10 h and up to 2 days, leaving a gap in information regarding the early dynamics of the interaction.

Despite being composed of only three organisms, the models discussed above exhibit emergent properties that are not observed in paired interactions as a result of what is described as Higher Order Interactions (HOIs). In such interactions, the effect of one competitor on another depends on the presence of a third species. These interactions arise with increased complexity in bacterial communities, and the presence of at least three organisms is required for HOIs to emerge. The term “higher-order interactions” has been defined in various ways in the ecological literature (Billick and Case, 1994). One use of the term HOI implies that indirect interactions arise, for instance, when the impact of one species on another requires or is modified by the presence of a third species (Wootton, 1994). HOIs have to be considered an intrinsic property of complex microbial communities. In describing collective behaviors, emergence refers to how collective properties arise from the properties of parts, how behavior at a larger scale arises from the detailed structure, and relationships at a finer scale. Emergent behaviors have been observed from large ensembles of elementary agents such as ant colonies and bird flocks. In ecology, emergence seeks to explain how biodiversity is maintained. The main question being to what extent the interaction between any two species is influenced by other species in the system.

Understanding higher-order interactions (HOIs) is crucial for comprehending the dynamics of communities and what allows species coexistence and makes them robust to the interactions between them and the environment (Grilli et al., 2017). In a higher-order interaction, one competitor modifies the competition between the other two (Levine et al., 2017). Although HOIs have been observed in bacterial communities, they were not explicitly described in some cases (Gallardo-Navarro and Santillán, 2016; Lozano et al., 2019; Piccardi et al., 2019). Nevertheless, there is a growing body of research on HOI dynamics in microbial communities (Mickalide and Kuehn, 2019; Sanchez-Gorostiaga et al., 2019).

In microbial communities, response to competition can be affected by various factors, such as growth conditions, life-history and functional traits, and interactions. However, communities are frequently disturbed by various factors, such as dispersion and invasion, which introduce new neighbors and require that their members be ready to respond to potential threats from competing organisms. The time of a response is an essential factor in competition, and a delay in responding, to antagonism, for instance, could lead to the death of a population. Nevertheless, immediate responses in competitive interactions have been the subject of limited research, as most laboratory studies on microbial interactions are conducted over periods of hours (Kerr et al., 2002; Kirkup and Riley, 2004; Vaz Jauri and Kinkel, 2014; Lozano et al., 2019), and in other cases, after days of interactions (Gallardo-Navarro and Santillán, 2016; Mickalide and Kuehn, 2019; Piccardi et al., 2019). Considering that the generation time of most aerobic, free-living bacteria is 20 to 60 min (albeit under laboratory conditions), the data from these experiments represent a late picture of an interaction that began within minutes of the bacteria’ first encounter. In this context, there is a need for models that allow us to study the immediate response to competition in bacterial communities.

In previous work, after studying a transitive interaction network of 78 bacterial species, a repeated pattern of Resistant (R), Antagonist (A), and Sensitive (S) species was observed that reflected a history of interactions in communities (Pérez-Gutiérrez et al., 2013). In the referred work, competitive interactions were evaluated in pairs, by recording halos of growth inhibition formed around a drop of a given species culture on a “mat” formed by another species extended on semi-solid medium. The result of the evaluation of 6,084 interactions, was a transitive network that resembled a food web. The interactions revealed three different ecological properties: “Antagonists,” “Sensitive” (sensitivity to the antagonists), as well as some non-connected “Resistant” species. A simulation with a cell automata model revealed that despite the antagonism no extinction of sensitive species occurred in a “structured environment” (Zapién-Campos et al., 2015).

Based on the above information where three interaction properties enable a complex natural microbial community, the hypothesis in this work is that a synthetic microbial community can be created using three members, each representing a different type of interaction (A, R, S). The simplicity of this community will allow the description of the assembly process and the exploration of the mechanisms underlying the paired and higher-order interactions. We anticipate that this higher-order community will exhibit emergent properties that cannot be observed in paired interactions. Specifically, we expect that the community will allow for the coexistence of sensitive and antagonistic strains, and that the sensitive strains will respond rapidly response to antagonistic interactions in order to survive. Additionally, we anticipate that the role of the resistant strain will only become apparent in the interaction of all three strains. In order to fully understand the dynamics of these interactions, we propose that evaluations must be conducted in minutes, even before the interacting bacteria have divided. This is a new approach that has not been attempted in previous works and will allow us to capture the early stages of community assembly. Through this work, we hope to shed light on the mechanisms that underlie the interactions between the different strains in the community, and how they contribute to the community dynamics.

To answer these questions, we constructed and evaluated a three-species synthetic community. Consisting of three Bacillota species each with a different characteristic: antagonistic (A), resistant to antagonism (R), and sensitive to antagonism (S). Each species had a distinct colony morphology that allowed simple quantification of the viability of each species in the interactions. The BARS community allowed us to observe emergent properties within minutes of the interaction, making it a unique model for exploring the immediate response to antagonism. The emergent property observed in BARS is the coexistence of the three different species, despite the antagonism observed in a paired interaction. BARS dynamics reproduce those of ecological communities: The effect of a strong competitor on the activity of its interactor, habitat modification, and changes in the abundance of the sensitive species. The BARS synthetic community model, with a simple experimental approach, has properties of a complex system with HOI dynamics.

## 2. Materials and methods

### 2.1. Species and media

The species chosen for the BARS community were *S. horikoshii* CH20a (*Sh*20a), *B. pumilus* CH145 (*Bp*145) and *B. cereus* CH111 (*Bc*111). We refer in the text to these species as sensitive (S), Antagonist (A) and Resistant (R), respectively, which are the ecological properties these species exhibit in the context of paired interactions. These were reported previously by Pérez-Gutiérrez et al. (2013) and were isolated from sediment in the Churince water system in the basin of Cuatrociénegas, Coahuila, México. The species were propagated in Marine Medium agar (MMs) and growth in liquid Marine Medium (MMl) at 28° C with constant shaking. Marine Medium composition can be found in Cerritos et al. (2011).

### 2.2. Assays for the evaluation of the interaction dynamics of the BARS community

Three species were selected that belonged to different species in the Bacillota phylum that had distinct phenotypes on Marine Medium (MM) agar plates. To determine the ideal proportion of each species for the interaction assays, the cell density of each species was evaluated by confrontations between the species directly in MM. To perform the interaction assays, overnight cultures were used (14 h). For the start of growth, 100 μL of the overnight culture was transferred to 30 mL fresh medium in 250 mL nephelometric flasks, and growth was monitored using absorbance on the Klett-Summerson colorimeter (red filter). Growth was monitored up to the exponential phase, and cultures were used in this phase. The interaction assays were started with a volume of 20 mL of S species with approximately 1.5 × 10^9^ CFU/ml, 4 mL of the A species with approximately 8×10^8^ CFU/ml and 4 mL of the R species with approximately 2 × 10^8^ CFU/ml. The ratio of 10:1:0.25 of the species S, A and R, were defined after a few trials of different cell numbers and proportions of A and S. The number of colonies that could be counted on plates limited some ratios. If larger proportions of the antagonist were used, the sensitive species CFU numbers could not be observed even at 5 min in interaction. We found that a ratio for S to A (1:10) allowed observing the dynamics of antagonism. We then observed that even a very low proportion of R cells allowed the survival of S in the triple interaction.

The interacting species were cultured in 50 mL Falcon tubes, in a final volume of 40 mL. MM was used to complete the volume of 40 mL for each interaction. Monocultures and different paired combinations and three species combinations were monitored. Samples were obtained every 5 min beginning at time 0 with a duration of the interaction typically up to 30 min. At each time point, dilutions of samples were immediately prepared in PBS and plated using glass beads to achieve a uniform distribution. Serial dilutions of samples through 1 × 10^−6^ were made and the highest three were plated on MM. The plated samples were incubated at 28° C for 48 h. CFU counts for each colony phenotype was obtained (S yellow, A white, R large white colonies). Dialysis membranes (Spectra/Pro® of 20 kDa pore) were used to evaluate interactions through diffusible metabolites (Master thesis Ortega-Aguilar, 2020). We did adjustments in cell density or volumes for some experiments, and these are explained for each assay. All interactions had at least three biological replicates. The mean data were plotted, with the standard deviation represented as a colored contour shadow to indicate the dispersion of the data (this was done using R-4.2.3).

### 2.3. Experiments to determine if the sensitive species culture contained a fraction of cells tolerant to antagonism before the interaction

To determine if some of the surviving colonies of the S species was the result of spores present in the cultures, an experiment was done using heat treatment. Heat treatment kills vegetative cells and allows survival of spores, that readily germinate when placed on MM. Monocultures of the S train and interactions of S.A and S:A:R were evaluated after 30 min and dilutions prepared and plated before and after heat-treatment of the sample at 70°C for 20 min. All interactions were tested using data from at least three biological replicates.

Another test to determine if there were S species tolerant to antagonism, consisted of looking por tolerant colonies of the S species growing within halos of inhibition around the antagonist species. A culture from the sensitive species was extended as a mat, and several drops of the antagonist species were placed as a drop on top of the mat (19 drops on five plates 100 × 15 mm, for a total 95 halos examined). Typically, since growth of the S species is prevented by the antagonists’ substance, a halo that extends a few millimeters from the growth of A was observed. If the S species had tolerant cells these would be expected to appear as satellite colonies within the halo of inhibition, or these would be completely clear in the absence of tolerant cells. As an example, four halos are shown in Supplementary Figure 3C.

### 2.4. Assays of supernatants (spent-medium) and lysates of *Bacillus cereus* CH111 to test their potential to protect the sensitive species from antagonism

Given that species R species can protect species S from the antagonism of A, even through a membrane, we decided to test if the supernatant or even the lysate of species R could have the same effect as the addition of the cell culture. To obtain culture supernatants, we followed the same procedure for culture conditions as in the interaction assays, at the same density of cells (2×10^8^ CFU/ml) and at the same point in the growth curve. Supernatants were centrifuged with a Sorvall Legend Micro 21R for 10 min at 13 rpm at room temperature and separated and filtered with a 45 μm pore Millex^Ⓡ^-GV membrane of PVDF (polyvinylidene difluoride) and nitrocellulose. For the experiments we substituted the culture of the R species culture with its supernatant and performed the experiment for the same 30 min with the species S and A. Samples were obtained every 5 min, and dilutions and plating were done as described above.

To obtain a CH111 lysate, we used the same conditions for the other cultures of species R and heated the culture for 30 min at 85°C to lyse the cells. The lysate was used as part of an assay with the species S and A for 30 min and samples were taken every 5 min. Dilutions and plating were done as described above.

## 3. Results

### 3.1. Design of the three-species BARS synthetic community

To create a synthetic community that could replicate the complexities of natural communities, we selected three species from a group of 78 that had been tested in a previous study in paired interactions. The strains had different “ecological roles” antagonistic (A), resistant (R), or sensitive (S) (Pérez-Gutiérrez et al., 2013). The selected strains were *Bacillus cereus* CH111 (R), *Bacillus pumilus* CH145 (A), and *Sutcliffiella horikoshii* CH20a (S) (Supplementary Figure 1). They could grow in the same medium with doubling times of 38, 51, and 30 min, for S, A, and R, respectively (Supplementary Figures 2A,B), which was relevant to establish the conditions to study their interaction dynamics. Finally, the unique colony morphology of each strain on plates allowed us to measure their viability during interactions (Supplementary Figure 2C). From now on, we will primarily use the abbreviations S, A, and R to denote the sensitive, antagonist, and resistant species, respectively.

### 3.2. Rapid dynamics and emergent property of BARS: sensitive species survival in a three-species community

**Figure 1:**
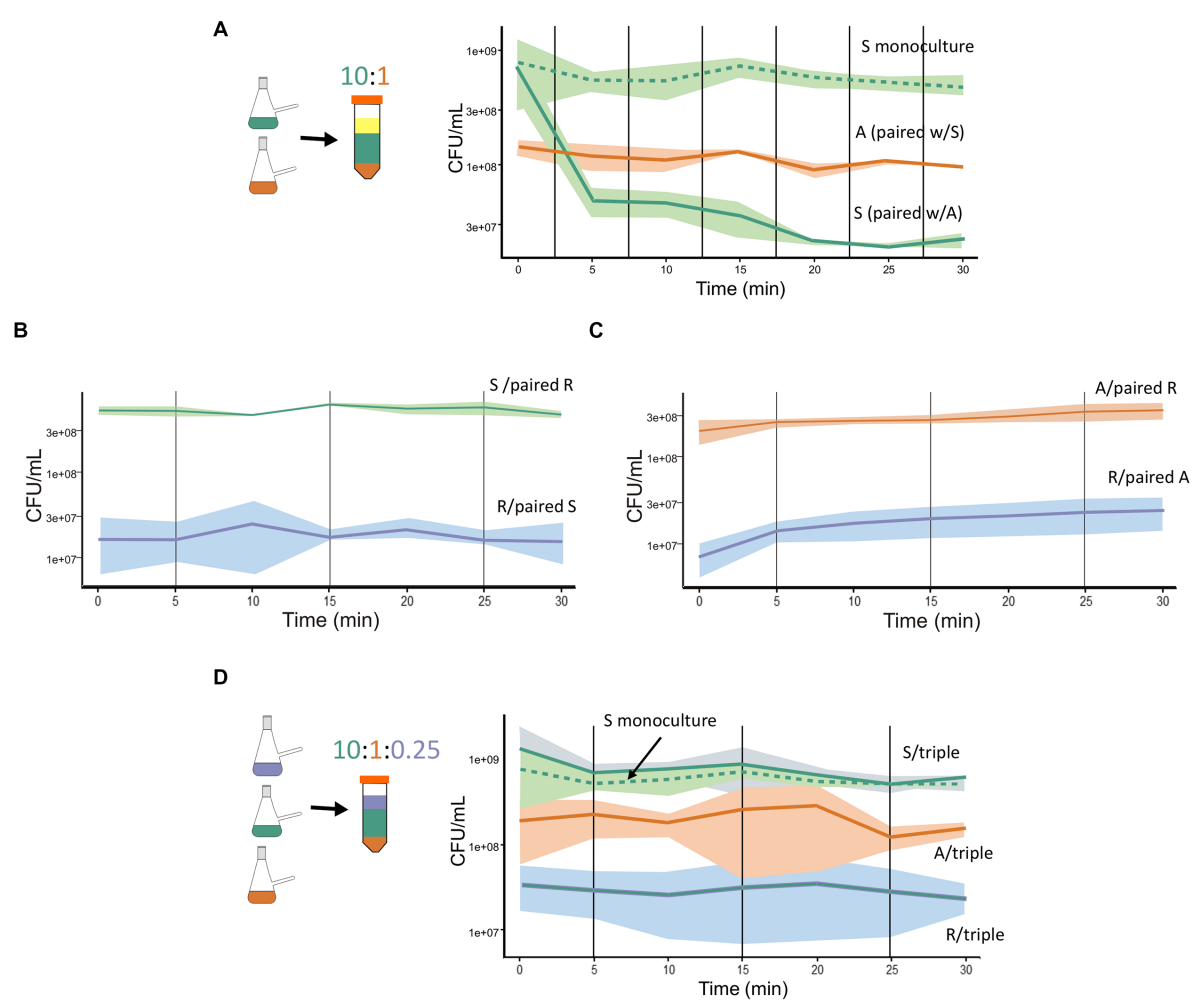
BARS synthetic community: from antagonistic paired interaction to emergent properties in a triple interaction. **(A)** Paired interaction between the S species (green solid line) and A species (orange solid line). The sensitive species maintained in monoculture (green dashed line) served as a control. **(B)** Paired interaction between S (green solid line) and R species (blue solid line). **(C)** Paired interaction between A (orange solid line) and R (orange solid line). **(D)** Triple interaction: R species (blue solid line), A species (orange solid line), and S species (green solid line). Graphs show 30-min kinetics of combined species that were mixed from monocultures (represented as nephelometric flasks) and proportions are indicated as layers of different colors (Falcon tubes). In the paired interaction, the proportion of S:A was 10:1. In the triple interaction, the proportion of S:A:R was 10:1:0.25. CFU for each species is plotted. The shadowed areas indicate the dispersion of data in three repetitions. The viable count of each species was obtained by plating at different time points after combining monocultures and counting the number of colonies of each morphology.

We designed experiments to assess the dynamics of antagonism in paired interactions of the A, R, and S species, with the expectation that the interaction dynamics would be fast, within 30 min. Our premise was that a rapid response from the sensitive species is crucial for its survival. We combined monocultures cultures in exponential phase, with the proportion of S to A species at approximately 10:1 (1.5 × 10^9^ CFU/mL and 8 × 10^8^ CFU/mL, respectively). We observed a rapid reduction in viable cell counts of S, with a decrease of over 60% within the first 5 min of interaction with the A species. The reduction continued at a slower pace over the next 15 min, with no further decrease by 30 min, suggesting stabilization of the dynamics (Figure 1A). In contrast, no antagonism was observed when paired interactions were set up between the S and R species or between the A and S species. In both cases, all species maintained the same initial viability after 30 min (Figures 1B,C).

The role of the R species in the three species community remained unclear. It showed no observable change in the dynamics with either the antagonistic or sensitive species in paired interactions. To investigate its role, we combined the A, R, and S species and evaluated their viability over time. Surprisingly, in the triple interaction, the S cell counts were not reduced despite the presence of the antagonist species (Figure 1D). Importantly, all three species maintained similar viability during the 30-min triple interaction, and their CFU counts remained stable in monoculture (R strain: 1.2 × 10^7^ and 2 × 10^7^ CFU/mL at times 0 and 30 min, respectively), while the antagonistic species maintained a mean CFU/ml of 1.5 × 10^8^ throughout the interaction (Supplementary Table 1). Overall, our experiments showed that paired interactions between BARS species A and S exhibit rapid dynamics of antagonism, resulting in a reduction in viable cell counts of the S species within 5 min. Notably, in the presence of R, the sensitive species in interaction with the antagonist survives. This is an emergent property that arose in the triple interaction, consistent of the immediate stability of the three-species with no observable antagonism. The role of the resistant species in this community appears to be the neutralization of the antagonistic interaction.

### 3.3. Tolerant community of S cells emerged as a result of the interaction with the antagonist

One possible explanation for the observed survival of 20% of the sensitive (S) cells in the presence of the antagonist (A) is that tolerant cells were present in the population before the interaction. To investigate this hypothesis, we designed assays to determine the presence of tolerant cells in the S species population prior to their interaction with the A species. To test for the presence of tolerant cells in the sensitive (S) species population, we conducted an assay in which the proportion of antagonist (A) cells was increased. If a fraction of tolerant cells already existed in the S culture, increasing the number of A cells would not affect the number of surviving S cells. Our results showed that when we increased the proportion of A cells from 1:10 to 10:10 in the assay, the number of cells killed by the antagonist also increased (see Supplementary Figure 3A). This result is consistent with the hypothesis that the tolerant community of S cells was not pre-existing. Interestingly, in both assays, the slope of killing slowed down after 15 min, and a stable tolerant community of S remained (Supplementary Figure 3A). In a second assay, we looked for surviving S colonies within the halo of antagonism produced by drops of the A culture on a mat of the S species on semi-solid medium. Consistent with our previous result, no surviving colonies were observed within the clear halos (Supplementary Figure 3B). Finally, we also investigated the possibility that spores present in the culture could be contributing to the tolerant population. *Bacillus* sporulation is typically triggered by nutrient starvation. However, since spores are temperature-resistant, we heat-treated a portion of the sample of the cultures after the interaction. No survival was observed in the heat-treated sample (Supplementary Figure 3C). Taken together, these results suggest that the observed tolerance of the S species to antagonism was not due to pre-existing tolerant cells and was instead the result of the interaction with the antagonist.

### 3.4. Antagonism and resistance in microbial interactions involve diffusible molecules

We aimed to investigate the mechanisms underlying the observed antagonism and resistance in the paired and triple interaction dynamics. Did antagonism require cell–cell contact or occur indirectly by secreting metabolites in the environment? The presence of an inhibition halo around the A species on a drop plate assay suggested the involvement of a diffusible molecule in the observed antagonism (see Supplementary Figure 3B). To investigate whether the observed dynamics could occur in the absence of direct cell–cell contact, we used dialysis membranes that permitted the exchange of metabolites while preventing physical interaction. We introduced the S culture into the membrane tubing and separated it from the antagonist species. Even with this physical separation, we observed antagonism between A and S, as shown in Supplementary Figure 4A. The inhibition of S was apparent from the beginning (CFU/mL 0 5.4 × 10^9^ to 1.1 × 10^9^ at times 0 and 10 min, respectively). We extended the duration of the assay by an additional 50 min to account for a possible diffusion delay, during which no further inhibition occurred. Additionally, we found that species R was able to safeguard the S cells even when separated from the A + S interaction (see Supplementary Figure 4B). These results demonstrate that the observed antagonism and protection involve diffusible molecules and do not require cell–cell contact.

### 3.5. Changing dynamics in the antagonism and neutralization of antagonistic metabolites in the media

**Figure 2:**
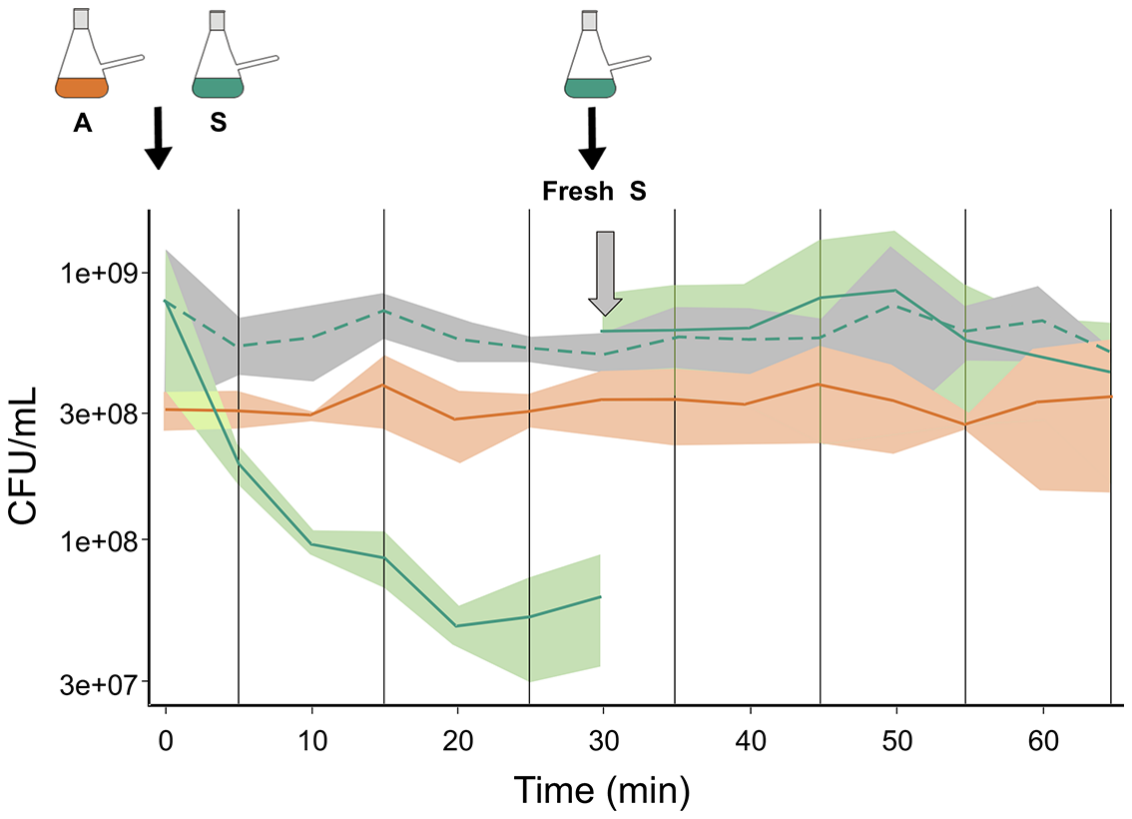
Changing dynamics: no antagonism was observed when fresh S cells were added to an ongoing paired interaction. A paired interaction of A and S was started from monocultures (depicted as nephelometric flasks). The first part of the graph shows the kinetics of the first 30 min of the paired interaction. S species (green solid line) and A species (orange solid line). The expected reduction in CFU of the sensitive species by antagonism was observed. Fresh S culture was added to the ongoing interaction after 30 min (depicted as Fresh S flask), and the viability of the species was monitored for another 30 min. After this time, no change in the viable count was observed for the S (green solid line). As a control, the CFU of the sensitive species in monoculture is shown (green discontinuous line).

Based on our earlier findings that antagonism was mediated by diffusible molecules and that the sensitive species did not experience further loss of viability after 20 min in paired interaction with the antagonist species, we investigated possible explanations. One possibility was that the antagonist metabolite was neutralized or destroyed, while another was that the antagonist species no longer antagonized the S species after some time. To assess these possibilities, we initiated an A:S interaction, and 30 min later we added a fresh culture of the S species. We allowed the interaction to proceed for another 30 min and found that during this second phase of interaction, the viability (counted as CFU) of the sensitive species did not decrease, indicating that the medium was not antagonizing and the antagonist species did not antagonize the freshly added S cells (Figure 2).

### 3.6. Investigating the change of state hypothesis of the sensitive species: pre-exposure to antagonism induces tolerance

**Figure 3:**
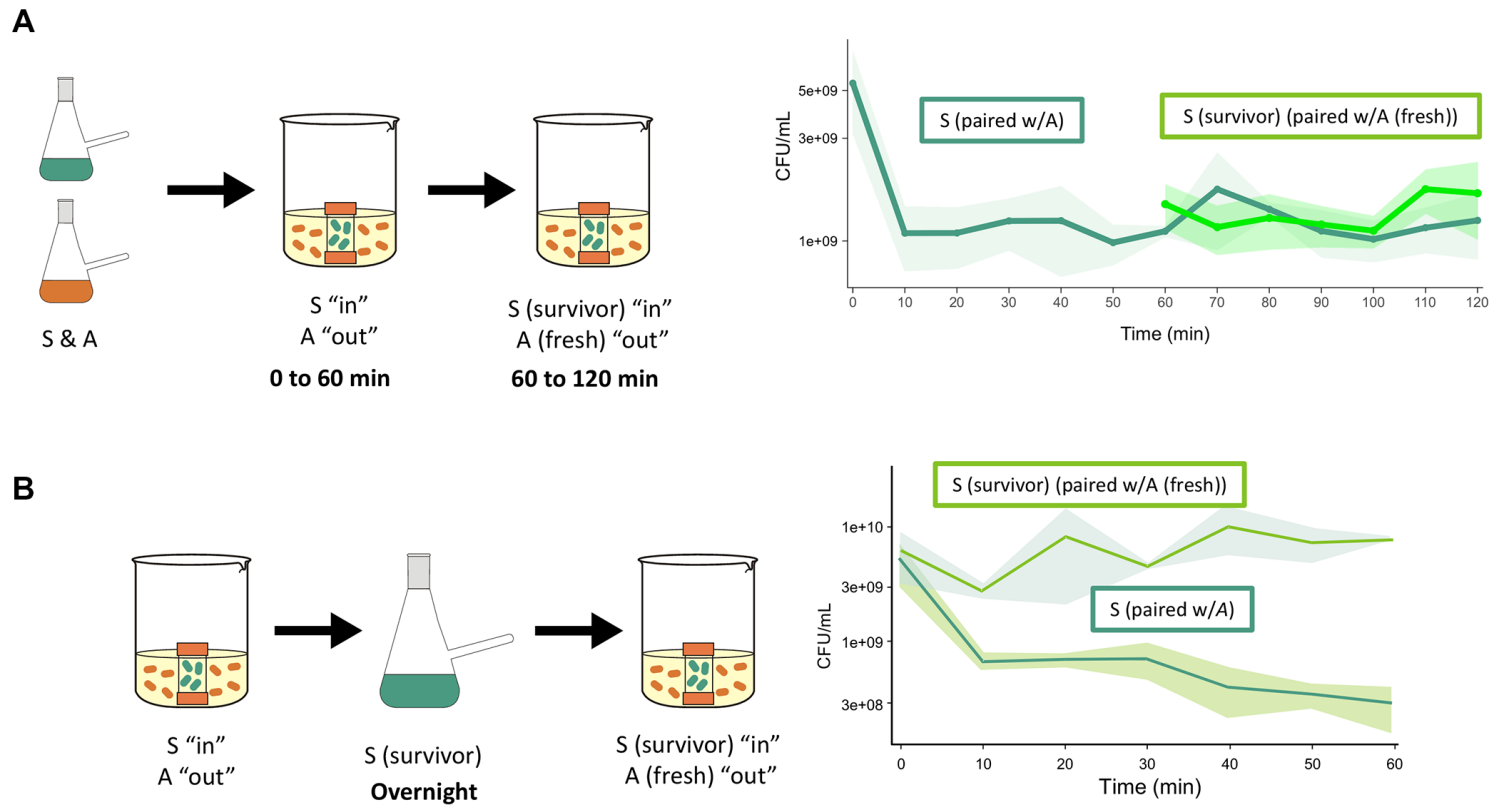
Acquired tolerance of the sensitive species after exposure to antagonist species. **(A)** A culture of *S species* was introduced into membranes that were exposed to the antagonist. After 60 min, membranes containing the sensitive species (surviving cells) were transferred to another beaker that contained fresh A cells. It can be observed that the CFUs of sensitive cells in the first interaction (dark green solid line) decreased in the first 10 min, but their viability did not change further through the next 50 min. The cells transferred in the membrane (survivors of the first interaction) did not decrease further upon interaction with fresh antagonis*t* (light green line). The control cells in membranes that were not transferred maintained the CFUs counts for 120 min (green discontinuous line). **(B)** Dynamics of S cells that survived antagonism and were grown overnight for a second interaction with the antagonist species. In the first interaction, the CFU of the S cells decreased (descending continuous green line). The survivor cells of the sensitive species (recovered from the membrane) were grown in monoculture (14 h) and challenged a second time. These cells tolerated a new confrontation with the antagonist (light green line).

After having ruled out the preexistence of tolerant S cells, we hypothesized that the surviving population resulted from induced tolerance after antagonism challenge. To test this hypothesis, we placed S cells inside a membrane and incubated them with A cells for 60 min. The membrane with the challenged S cells was then transferred to a flask with a fresh culture of A cells. The membrane-isolated S cells showed increased survival, suggesting that exposure to A induced tolerance (Figure 3A). We then investigated whether survivor S cells could maintain tolerance if grown overnight, so the challenged cells were used to start a culture. Figure 3B shows a reduced antagonism effect from A cells, indicating that the tolerance could be sustained, albeit at a reduced level, after cell division.

One possible explanation for the survival of a fraction of S cells exposed to A cells is that the concentration of the antagonistic compound was not lethal to the entire population, allowing some cells to activate genes that conferred tolerance. This tolerance phenotype was maintained even after overnight growth, as shown in Figure 3B. This finding is consistent with the absence of tolerant cells from the start of the experiment. However, only 20% of cells were sensitive, indicating that tolerance was partially retained. We consider this a “change of state” in which the S species becomes tolerant to the A species within 15 min in interaction, another emergent property in the BARS dynamics. This change of state is another indication of the complex and dynamic nature of this synthetic community.

### 3.7. Only live cells of R provide full and immediate protection to S cells from antagonism

To understand how the R species prevents the S species from being antagonized by A, we conducted a series of experiments. We previously observed that the R species could protect the S species even when separated with a dialysis membrane. This suggested that protection could be a passive process mediated by molecules in a lysate or filtered supernatant (spent medium). However, when we evaluated whether a heat-treated lysate of R cells could protect the sensitive cells from antagonism, we found that it did not fully eliminate the antagonistic effect of A over S (75% of S cells were killed). We then evaluated whether filtered A resistant culture supernatant could neutralize antagonism and found that it did provide some protection, as the population of S cells decreased at a slower rate compared to the interaction between only S and A. However, only the live R cells conferred the full and immediate protection observed in the triple interaction. These results suggest that the mechanism of protection of the resistant species is a combination of molecules already expressed and found in the lysate, but may also require the expression of a possible metabolite during and as a result of the interaction. Our findings highlight the complex interplay between the different species and the potential involvement of multiple mechanisms in conferring protection against antagonism.

### 3.8. Small changes in the proportion of R in the triple interaction have a significant impact on system behavior

**Figure 4:**
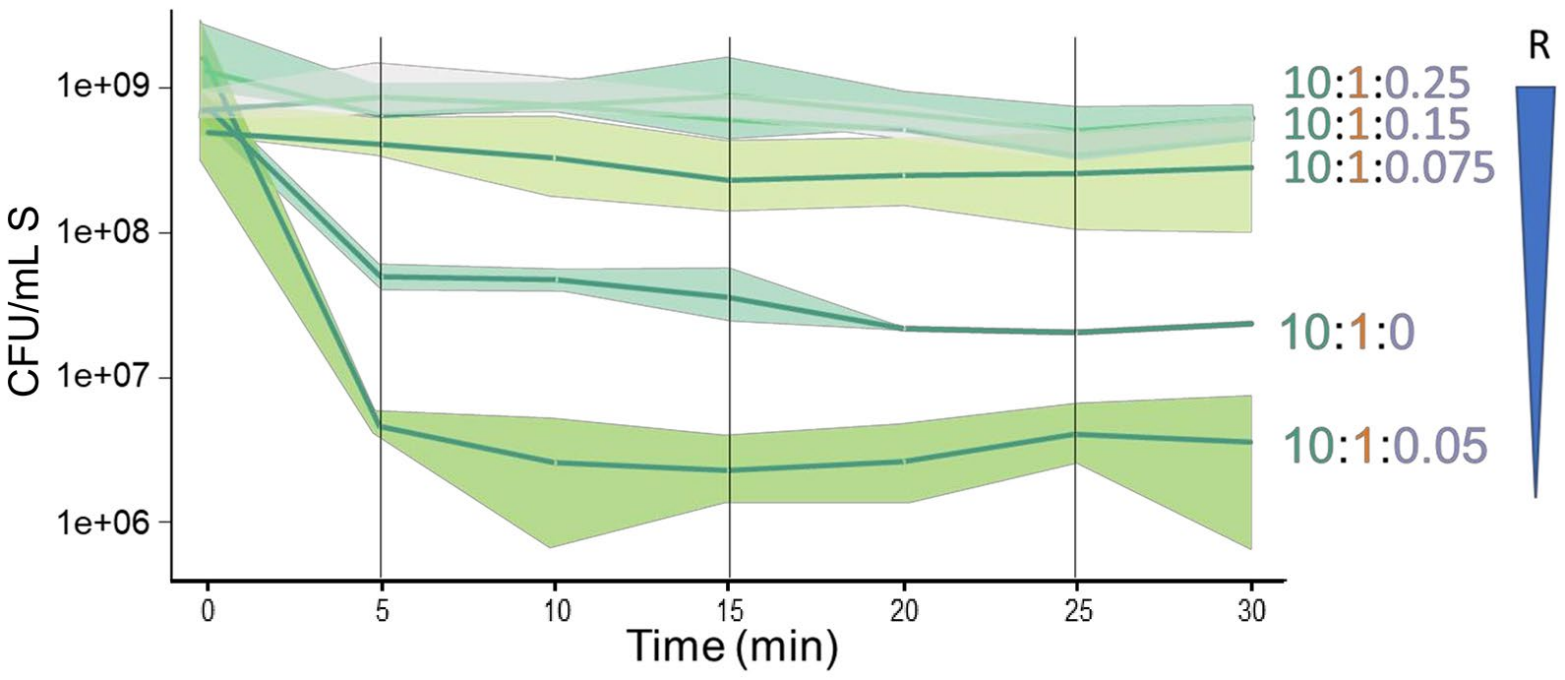
The dynamics of the sensitive species is highly sensitive to a decrease in the proportion of the resistant species in a triple interaction. Triple interactions were evaluated reducing the proportion of the R cells relative to the S and A cells (schematized by the inverted blue triangle). Starting with 10:1:25, with R at 2 ×10^8^ CFU/ml, the sensitive species is not antagonized in the presence of the resistant species. Upon sequential reduction from 1.3 ×10^8^ CFU/ml (10:1:0.15) and 6.6 × 10^7^ CFU/mL (10:1:0.075), a slight decrease of the population of the sensitive species is observed. Unexpectedly, a reduction to 4 ×10^7^ CFU/ml (10:1:0.05), resulted in a large reduction in the population of the sensitive species, an effect even more dramatic than in a paired interaction of just the sensitive and antagonist species (10:1:0).

We conducted experiments to investigate the importance of the proportions of sensitive, antagonistic, and resistant species in their interaction. It was intriguing that even a small proportion of R cells in the triple interaction could protect S from antagonism. The proportion used was 10:1:0.25 (S:A:R, respectively). To determine the minimum R cell density needed for protection, we tested changes in the abundance of the resistant species, maintaining the proportion of S:A at 10:1 and lowering the proportion of R to 0.15 (1.3 × 10^8^ CFU/ml) and 0.075 (6.6 × 10^7^ CFU/ml). We found that a proportion of 0.15 still protected S, while 0.075 led to decreased protection (Figure 4). However, when the proportion of R was decreased to 0.05 (4 × 10^7^ CFU/ml), an unexpected negative effect on the sensitive species was observed, with an increased antagonism of S that was even higher than that observed in the paired interaction between S and A (10:1:0 in Figure 4). These findings suggest that the cell density of R plays a critical role in determining the fate of S cells when exposed to A antagonism and that there is a non-linear dynamics in the interaction.

## 4. Discussion

In this work, we present a three-species synthetic community that we named BARS, consisting of three Bacillota species characterized as the antagonist (A), resistant to antagonism (R), and sensitive to antagonism (S). The community exhibits endogenous dynamics that provide clues as to how complex systems arise from the interaction of a few components allowing species to coexist in antagonistic interaction. The emergent property observed in BARS is the coexistence of the three different species, despite the antagonism observed in a paired interaction. Microbial communities exhibit complex adaptive system behavior, and the BARS model, exemplifies an HOI community. It displays emergent states and suggest non-linear dynamics. The BARS model achieved a stable, coordinated system immediately after starting a triple interaction assay. The system’s emergent stability was achieved by changing the “rules” of antagonism to a non-antagonistic mode, resulting in endogenous and higher-order properties that were not predictable from either the examination of individual members or the paired interactions.

**Figure 5:**
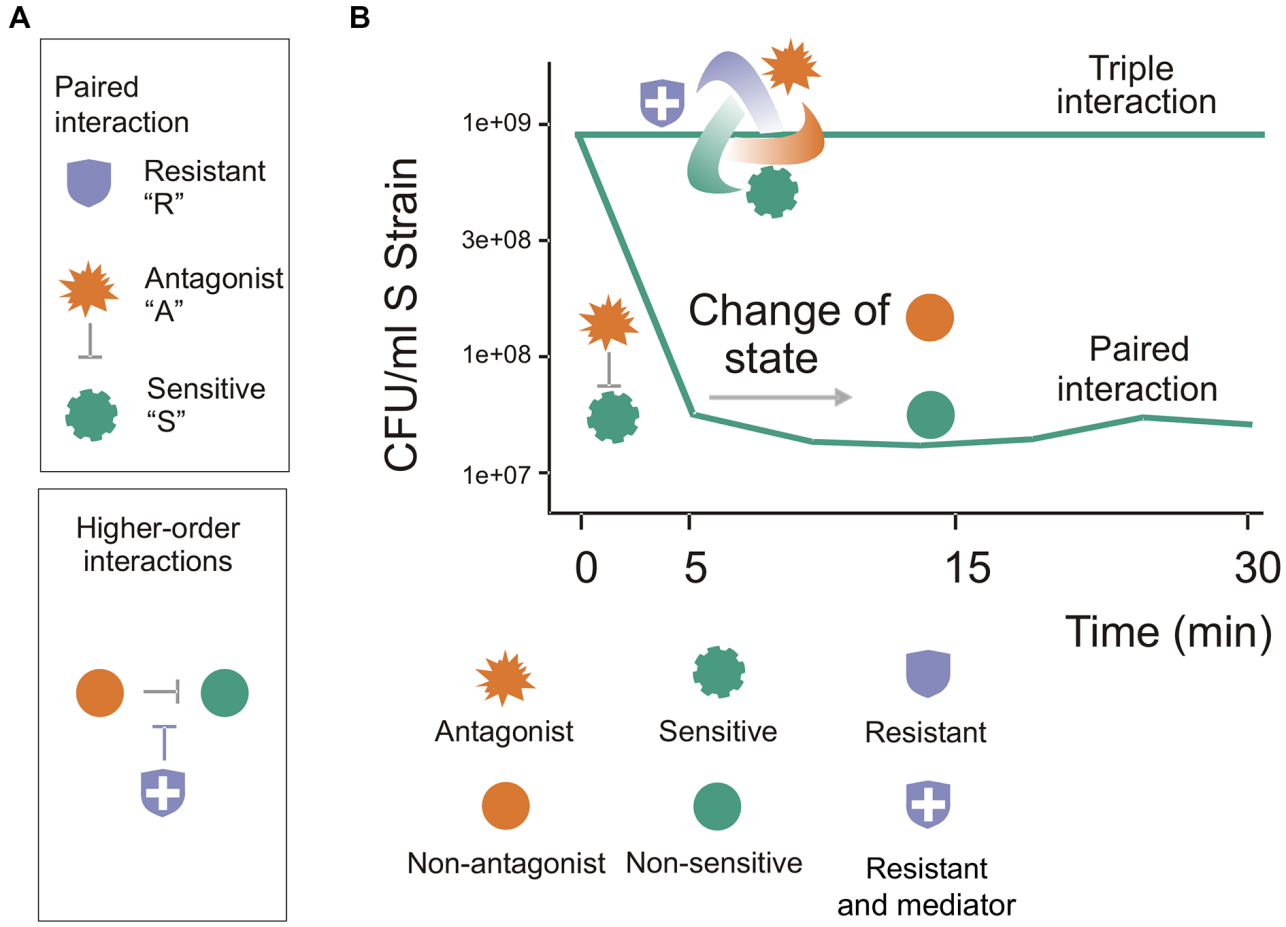
The three-species BARS synthetic community: a model of a complex system with emergent properties. The BARS synthetic community serves as a model of a complex system with emergent properties. **(A)** Each species exhibits known properties from paired interactions, but their behavior in a triple community cannot be predicted. **(B)** As a higher-order interaction system, it adopts features of complex communities, with the resistant species acting as a mediator (blue shield symbol). During paired interactions, a strong antagonism occurs in the first 5 min, but no further antagonism is observed in the next 20 min (the curve decreases and then remains stable). Notably, after 30 min, a qualitative change is observed, as the A species no longer antagonizes S cells, and S cells become tolerant to A. The top line in the figure shows that a stable three-species community emerges and antagonism is not observed, suggesting that an immediate dynamic takes place.

The essential feature of the BARS model is its rapid response to interaction, occurring within 30 min. In this model, during a paired interaction, the sensitive species population survived antagonism and became tolerant and the A species cease to antagonize. In the triple interaction the R species promoted the survival of the sensitive species. The BARS three-species model, summarized in Figure 5, captures the properties of complex communities with highly interactive organisms that engage in metabolic coupling, promote endogenous dynamics, and form self-organizing structures. The results suggest mechanisms of negative interference competition mediated by antibiotics and antibiotic resistance genes. We show that the survival of the sensitive strain was due to the induction of tolerant cells during interaction, and that antagonism was caused by diffusible antimicrobial compounds. We also observed that the detoxification of the medium and degradation of antimicrobial substances, and that the antagonism and protection offered by the resistant strain were density-dependent.

The three BARS species were selected based on specific criteria, including their distinct ecological “roles” assigned in a previous work, where paired interactions in a 78 × 78 species matrix were evaluated (Pérez-Gutiérrez et al., 2013). The species are part of a collection from five sampled communities for which antagonism interaction networks within each community have been described. Within each of the five networks, less antagonism was observed than in the combined network. This suggests that there may have been a history of community interactions, in which species that were able to tolerate one another were enriched within each community. As such, it is possible that these bacteria have acquired particular antibiotic production genes and that an “arms race” has been playing out, by which these bacteria have evolved multiple forms of antibiotic resistance. The genetic repertoire for exerting antagonism, inducing resistance, and detoxification capacity may have been acquired and fine-tuned through their evolution in their sediment communities. Our own research has shown intraspecies variation in *Bacillus* strains co-occurring in the sediment communities of the Churince system in Cuatrocienegas, Mexico. We have shown that these strains exhibit high levels of intraspecific phenotypic and genotypic diversity (Gómez-Lunar et al., 2016; Rodríguez-Torres et al., 2017), indicating a microevolutionary process that optimizes their ability to respond to environmental changes and microbial interaction challenges.

Communities experience invasion continuously, or their members get shuffled around to find themselves with new neighbors. They have evolved adaptations to respond immediately to biotic and abiotic challenges in complex communities, making them experts in integrating fine-grained information and collectively finding solutions that we observe as emergent phenomena. Their capacity to respond immediately was probably shaped by the history of past environmental states. As Flack (2017) notes, biological systems make hypothesis about the present and future environments they or their offspring will encounter based on the history of past environmental states they or their ancestors have experienced. The importance of BARS and other models in synthetic ecology is that they allow us to address questions regarding the assembly of communities, a process where biotic and abiotic filters define the diversity and abundance of species. Given the complexity of this endeavor, having models that reduce the number of individuals and environmental variables is essential. However, equally important and missing in most microbial models, is being able to address the immediate consequence of the invasion of a community by new individuals. Different statistical and information theory methods can be used to detect causal relationships. Herrera Paredes et al. (2018) investigated the predictive performance of various statistical models. They demonstrated that it is possible to infer causality between microbiome composition and host phenotypes in complex systems.

We speculate that within the complex systems, the BARS model behavior may represent a case of “explainable” weak emergence. Weak emergence describes the way in which complex systems exhibit new properties or behaviors that are not present in their individual parts but can be explained in terms of the interactions between the parts. To test this hypothesis, experiments could be conducted to determine whether the emergent features in the triple interaction are simply an extension of the mechanisms already observed in the paired, lower-level interaction. This investigation would require an examination of the molecular mechanisms of antagonism and resistance. Various strategies for antagonism have been reported, including the secretion of enzymes or metabolites into the extracellular space or through outer membrane vesicles (OMVs; Manning and Kuehn, 2011; Kulkarni et al., 2015; Liao et al., 2015; Lekmeechai et al., 2018; Roszkowiak et al., 2019). Toxin production is a common response to competitive interactions, but factors such as species frequency, nutrient level, relatedness, and the cost of toxin production all influence bacterial decision-making (Niehus et al., 2021).

Another property of the dynamics of the BARS community is the induced tolerance of a fraction of the sensitive species population to antagonism. Our results suggest that under the conditions evaluated, the observed antagonism occurred during the interaction and there were no preexisting tolerant cells, but rather the cells that survived are those that induced resistance to antagonism. This resembles antibiotic resistance induction that has been extensively studied in bacteria, with some examples specifically in *Bacillus* (Chancey et al., 2012). The mechanism of defense can include enzymes that degrade antibiotics or toxins. The detoxification of the environment by one of the interactors in a community has been described. The idea of a third interactor as a mediator of antagonism was reported by Kelsic et al. (2015). They proposed that the antibiotic-resistant species in a community have the function of degrading the toxins and antibiotics in the environment, allowing a sensitive species to cohabit in the community with the antibiotic producer species. In studies on antibiotic resistance, detoxifier capacities have been attributed to species able to express enzymes that can degrade antibiotics, such as β-lactamases. In a gram-negative model, the presence of β-lactamases was reported as a modulator of an antagonistic interaction by the cleavage and deactivation of antibiotics (Medaney et al., 2015; Frost et al., 2018). We speculate that the R species can neutralize or detoxify the environment from the antagonist substance produced by the antagonist species.

Microbial models often fail to capture the immediate consequences of new individuals invading a community, a crucial aspect akin to missing challenges and conflicts in a story plot and only finding out how the story concludes. In the BARS experimental model, by assessing the dynamics at 5 min, the strong antagonism and immediate responses between the paired interactions of A and S were observed, as well as the seemingly instantaneous emergent properties in the triple interaction of A, R, and S, that allowed the three species to coexist. Understanding these early stages of community formation is critical for predicting long-term dynamics and the development of stable, functional communities. The rapid molecular responses in bacteria can explain these rapid responses. *B. subtilis* serves as an example of a rapid defensive response against antibiotics. Cao et al. (2002) showed that in *Bacillus subtilis*, Extracellular Sigma Factors (ECF) coordinate the defensive response within 3 to 10 min, specifically in response to antibiotics that inhibit cell wall synthesis. ECF sigma factors help maintain cell envelope integrity by activating the expression of genes that inactivate or detoxify antibiotics or alter cell surface properties. Therefore, a quick response by the bacterial population through their defense mechanisms is crucial for survival.

This rapid response in BARS suggests that the response is regulated and has important implications for understanding natural communities. Niehus et al. (2021) modeled the competition of species and the effect of a regulated versus a constitutive production of a toxin. They found significant benefits of regulation of timing as this minimizes cost and maximizes the effect on an opponent. They suggest that this is similar to classical predictions from the game theory of animal combat and that the regulation of combat in bacteria is about timing an attack and turning off defenses once the attack is over (Niehus et al., 2021). To address timing and gene expression in synthetic communities, it is exciting the significant advancements in microfluidic analysis. These devices offer single-cell analysis, cell–cell interactions and growth dynamics with high spatio-temporal resolution, allowing for the control of environmental conditions (physical, biological, and chemical stimuli) in a high-throughput manner (reviewed in Burmeister and Grünberger, 2020).

HOI can be observed in studies of synthetic communities of microorganisms reported in recent years (Abrudan et al., 2015; Gallardo-Navarro and Santillán, 2016; Lozano et al., 2019; Mickalide and Kuehn, 2019; Piccardi et al., 2019), some of them showing some properties similar to the BARS community dynamics. Gallardo-Navarro and Santillán (2016) described a model in which a resistant species (*Staphylococcus* sp.) seems to protect a sensitive one (*Bacillus. aquimaris*) from the antagonism of a *B. pumilus* species. Another example comes from the work of Lozano et al. (2019), in a model named THOR (the hitchhikers of the rhizosphere), with a similar three-species community; interestingly, in this model, the resistant species is also *B. cereus* that protects a species of *Flavobacterium johnsoniae* from growth inhibition by *Pseudomonas koreensis.* Given the emergent properties that we and others have observed, maybe we have to carefully evaluate more data before applying a “rule” such as the one proposed by Friedman for the prediction that species that all coexist with each other in pairs will survive, whereas species that are excluded by any of the surviving species will go extinct (Friedman et al., 2017). Since complex network systems with non-linear dynamics are capable of self-organization, characterized by feedback, stability, and hierarchy, and such systems display new arising or emergent features, we do not think that paired interactions can predict the stability or survival of a species.

One of the key features of complex systems is that they often exhibit non-linear dynamics, making them more susceptible to sudden transitions or changes in behavior. In BARS, the ratio of resistance cells relative to those of the antagonist and sensitive species defines whether the antagonistic interaction is subdued or increased. In the report from Medaney et al. (2015), they observed an increase in the cell density of the interactors as an emergent property since the population of the sensitive species was positively correlated with the cell density of the resistant one. It was intriguing that protection from the R species occurred when its proportion relative to the antagonist was small (10:1:0.25) but when the cell density of the resistant species was lowered (10:1:0.05), the sensitive species seemed to be more affected by the antagonist. The density experiment with the resistant species surprised us, as a slight decrease in the ratio of R seemed to send the system in the opposite direction. Since the BARS model dynamics are very sensitive to the initial species cell ratio, suggesting non-linear dynamics, experiments at different densities and frequencies of species A, S, and R are needed for the mathematical modeling of the interaction. Understanding the rules governing microbial community dynamics over time is still limited. However, mathematical modeling has emerged as a valuable tool in this field. For example, Coyte et al. (2015) employed a mathematical approach to model microbial communities. They found that high species diversity is more likely to be stable when competitive rather than cooperative interactions dominate the system. The principles gathered from studying such a model will allow to describe the interaction between components of the system, to build models that test predictions and increase our understanding of microbial community dynamics.

Although they are intended to predict community dynamics, scaling models of synthetic ecology with culturable microorganisms can be challenging. In the study of human gut communities, some research groups are working on building increasingly large synthetic communities (currently up to 100 species) (van Leeuwen et al., 2023), and mathematical modeling is being used to predict the metabolic behavior of gut communities. Metagenomic and transcriptomic approaches are now commonly used to study microbial communities at large scales and over time (Taş et al., 2021). For instance, using metagenome and metatranscriptome analysis, Chuckran et al. (2021) demonstrated that soil microbial communities respond to carbon inputs within hours by altering gene expression. Despite the difficulties in replicating complex natural ecosystems in laboratory settings, numerous metagenomic studies have been conducted to investigate gut communities, water treatment plants, and industrial bioreactors, among others. Mathematical models are employed to better understand how community composition and emergent functions translate to natural systems (van den Berg et al., 2022).

Given the simplicity of the BARS community, transcriptomics analysis will allow identifying genes involved in the immediate response to community interactions. The results will aid in the identification of substances involved in tolerance and antagonism, and may reveal if there is a connection between the dynamics of the paired and the triple interaction.

## 5. Conclusion

The BARS three-species synthetic community serves as a valuable higher-order model, providing clues to important ecological questions. For instance, it helps to understand how complex systems arise from different components and the role of microbial interaction dynamics in promoting species coexistence. At higher-order levels, the bars model demonstrates the emergence of coexistence between sensitive and antagonistic species, where antagonistic competition is no longer observed. Of great significance is that the BARS model allows for evaluating the immediate response of bacteria in antagonistic competition. This contrasts to most studies, which evaluate community assembly over hours or days, leaving the initial encounter between community members as a black box; we miss the game’s climax and are left only with a picture of the winners. The BARS synthetic community model simplifies the study of dynamic interactions but maintains emergent features of ecological communities.

## Data availability statement

The original contributions presented in the study are included in the article/Supplementary material, further inquiries can be directed to the corresponding author.

## Author contributions

GO-Á conceived the project and interpreted data and results. BA-S conducted the experiments and analyzed and interpreted data. BA-S and GO-Á contributed to writing the manuscript.

## Funding

This project was financed by Conacyt Ciencia de Frontera No. 39589 to GO-Á. BA-S acknowledges fellowship from Conacyt.

## Conflict of interest

The authors declare that the research was conducted in the absence of any commercial or financial relationships that could be construed as a potential conflict of interest.

## Publisher’s note

All claims expressed in this article are solely those of the authors and do not necessarily represent those of their affiliated organizations, or those of the publisher, the editors and the reviewers. Any product that may be evaluated in this article, or claim that may be made by its manufacturer, is not guaranteed or endorsed by the publisher.
