# Supplementary material for: A three-species synthetic community model whose rapid response to antagonism allows the study of higher-order dynamics and emergent properties in minutes
Source: Front Microbiol. 2023 Jun 2;14:1057883. doi: 10.3389/fmicb.2023.1057883 (PMC10272403; doi:10.3389/fmicb.2023.1057883)
Supplement: Supplementary file 1 [file Data_Sheet_1.pdf]

Supplementary figure footnotes

Supplementary Figure 1. Species selection for the BARS synthetic community. Based on a past evaluation by Pérez-Gutiérrez et al (2013), the phenotype on paired interaction was re-evaluated on a mat from a *Sutclifiella horikoshii* 20a culture (sensitive species, S). Species *Bacillus pumilus* 145 was selected as the antagonist of species (A), and *Bacillus cereus* 111 as a non-antagonist and resistant species (R) (resistance was evaluated in a different experiment).

Supplementary Figure 2. Duplication time and morphology of colonies of the BARS species A. All species were grown on Marine Medium. Their doubling times were 30, 38 and 51 min for the R, S and A cells, respectively. B. In-degree refers to the number of species antagonized, and out-degree is the number of species that a given species antagonized (total being 78). Based on data obtained from the network from paired antagonism with 78 species (Pérez-Gutiérrez *et al.*, 2013). C and D. The BARS species exhibited different colony morphologies that allowed their individual quantification as CFU in a mixed plating. Plates in C show colonies after a 5 min paired interaction between antagonist and sensitive species. A zoom out facilitates observing the different phenotypes of the colonies, and particularly the survival of the S strain (yellow) always separated from the A strain (whitish). Plates in D show the results after 5 min in a triple interaction between A (white, medium-sized colonies), S (yellow), and R (large white colonies).

Supplementary figure 3. **Tolerance is induced from the interaction with the antagonist and does not preexist in the sensitive population**. A) A paired dynamic assay was done with two different proportions of S and A species. Little survival of the population of S was observed in the 10:10 compared to the 10:1 confrontation, thus showing that the observed tolerant cells in 10:1 were not preexisting, as a higher number of A cells (10:10) drastically reduced the number of surviving S cells. The control, without A, remained stable (discontinuous line). B) Clear halos around the colonies of A over a mat of S do not show any satellite colonies of S capable of growing within the inhibition zone, suggesting that there are no preexisting S cells that were tolerant to antagonism. C) After a 30 min dynamics of the S species in monoculture, the paired S:A interaction, or the triple S:A:R interaction, dilutions were plated either directly or after heat treatment at 80° C for 30 min, to which only spores survive. No colonies were observed in the heat-treated samples. Tolerance to antagonism of S cells, therefore, is not explained by preexisting spores.

Supplementary figure 4. Dynamics of the BARS interaction can be reproduced through a membrane and are thus independent of cell-cell contact. A. Antagonism and protection from antagonism in the BARS model occur through metabolites diffusion. A culture from the S species was placed inside a membrane and the membrane placed in a beaker containing an A species culture. Aliquots from each were plated at different times to determine CFUs. The S species was antagonized by A in the absence of cell contact (green solid line). **B.** The R species culture stabilized the antagonistic interaction without cell contact. A culture of the R species was placed out of the membrane and the sensitive and antagonist cells were both inside the membrane. CFUs o each S, A and R are shown by green, orange, and blue lines, respectively.

Supplementary figure 5. Reduction of antagonism in the A/S interaction by an R species cell lysate or supernatant. A. Addition of a lysed culture of the resistant species to a paired interaction between A and S species reduced antagonism. The A and S kinetics are shown by the orange and green lines, respectively. The sensitive species in monoculture (no lysate added) was evaluated as a control (green discontinuous line). **B.** A filtered supernatant from an R species culture (spent-medium) was added to a paired interaction between A and S species. The A and S kinetics are shown by the orange and green lines, respectively. As a control, the sensitive species in monoculture with no supernatant added (green discontinuous line). Both the lysate and supernatant reduced and retarded the antagonism of A over S.
