# Supplementary figures and images for: A three-species synthetic community model whose rapid response to antagonism allows the study of higher-order dynamics and emergent properties in minutes
Source: Front Microbiol. 2023 Jun 2;14:1057883. doi: 10.3389/fmicb.2023.1057883 (PMC10272403; doi:10.3389/fmicb.2023.1057883)

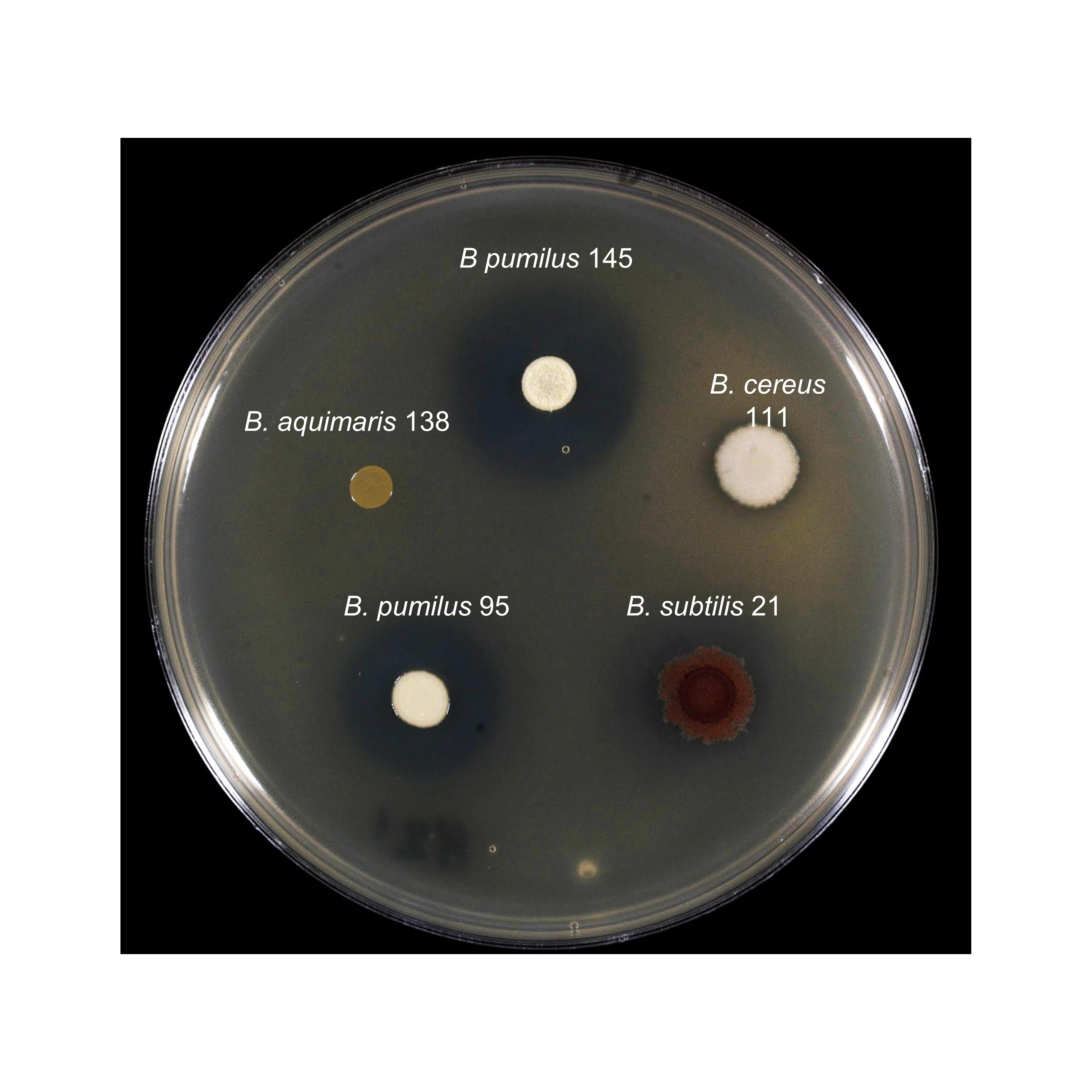

Supplement: Supplementary file 2 [file Image_1.tiff]

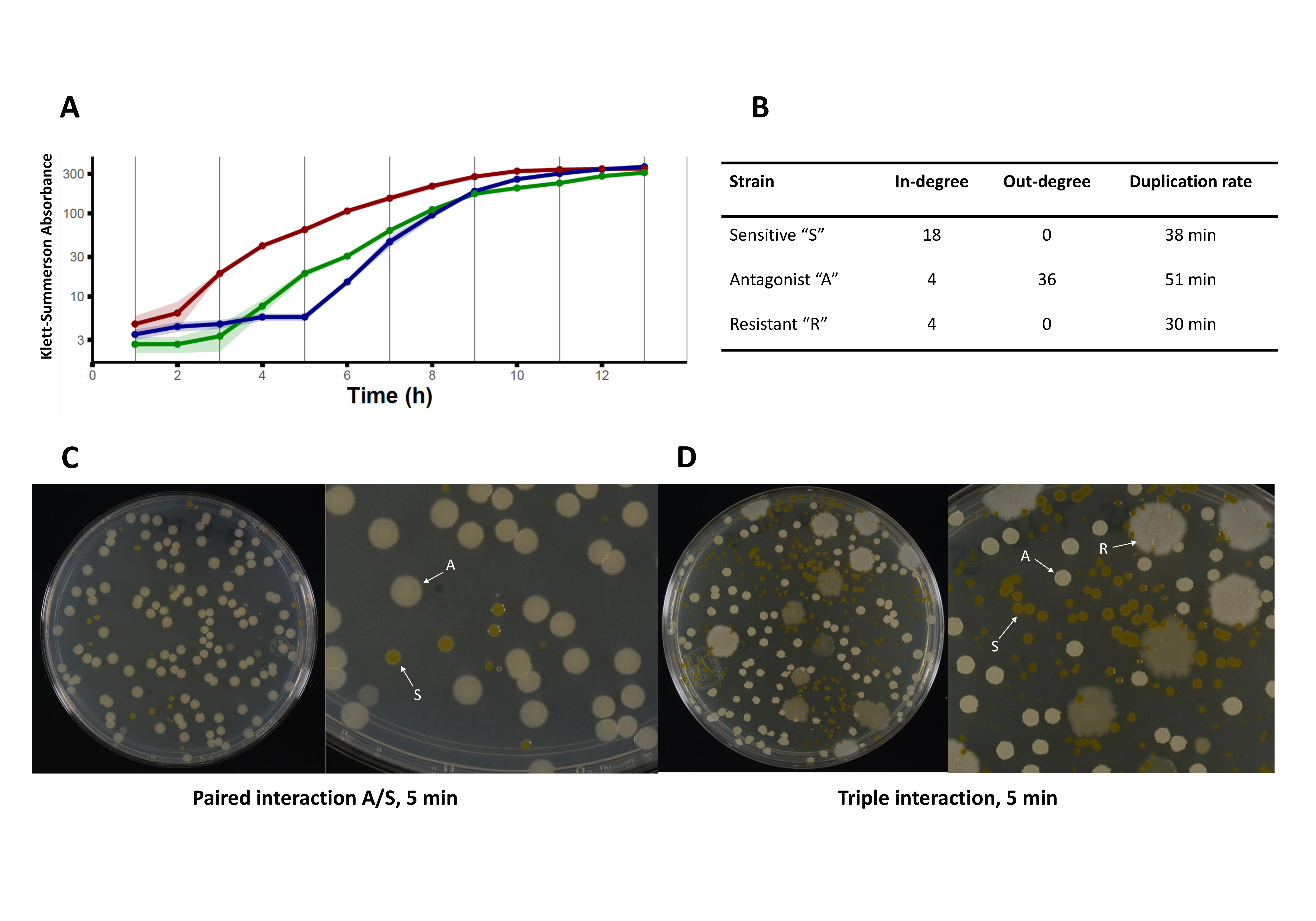

Supplement: Supplementary file 3 [file Image_2.tiff]

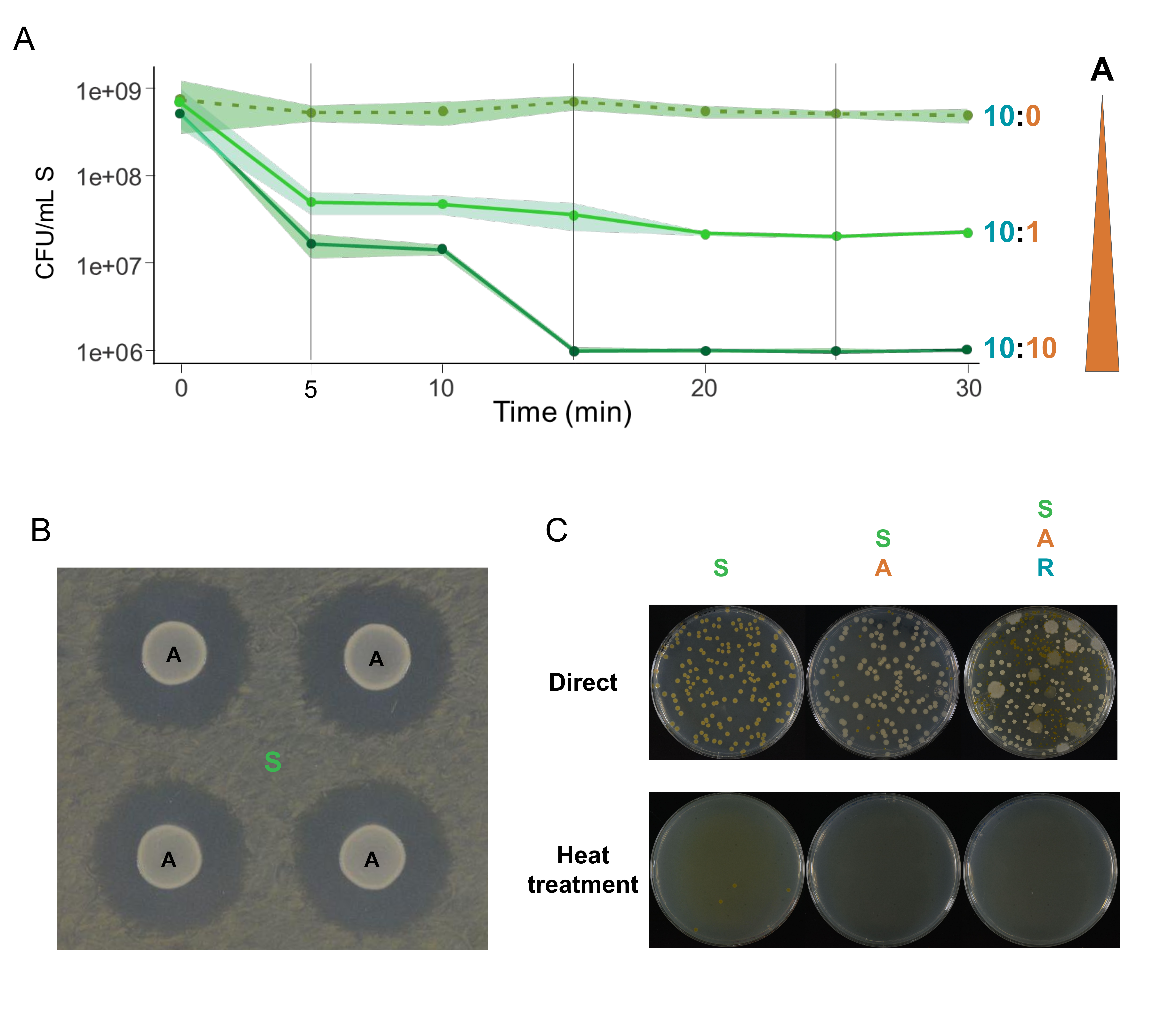

Supplement: Supplementary file 4 [file Image_3.tiff]

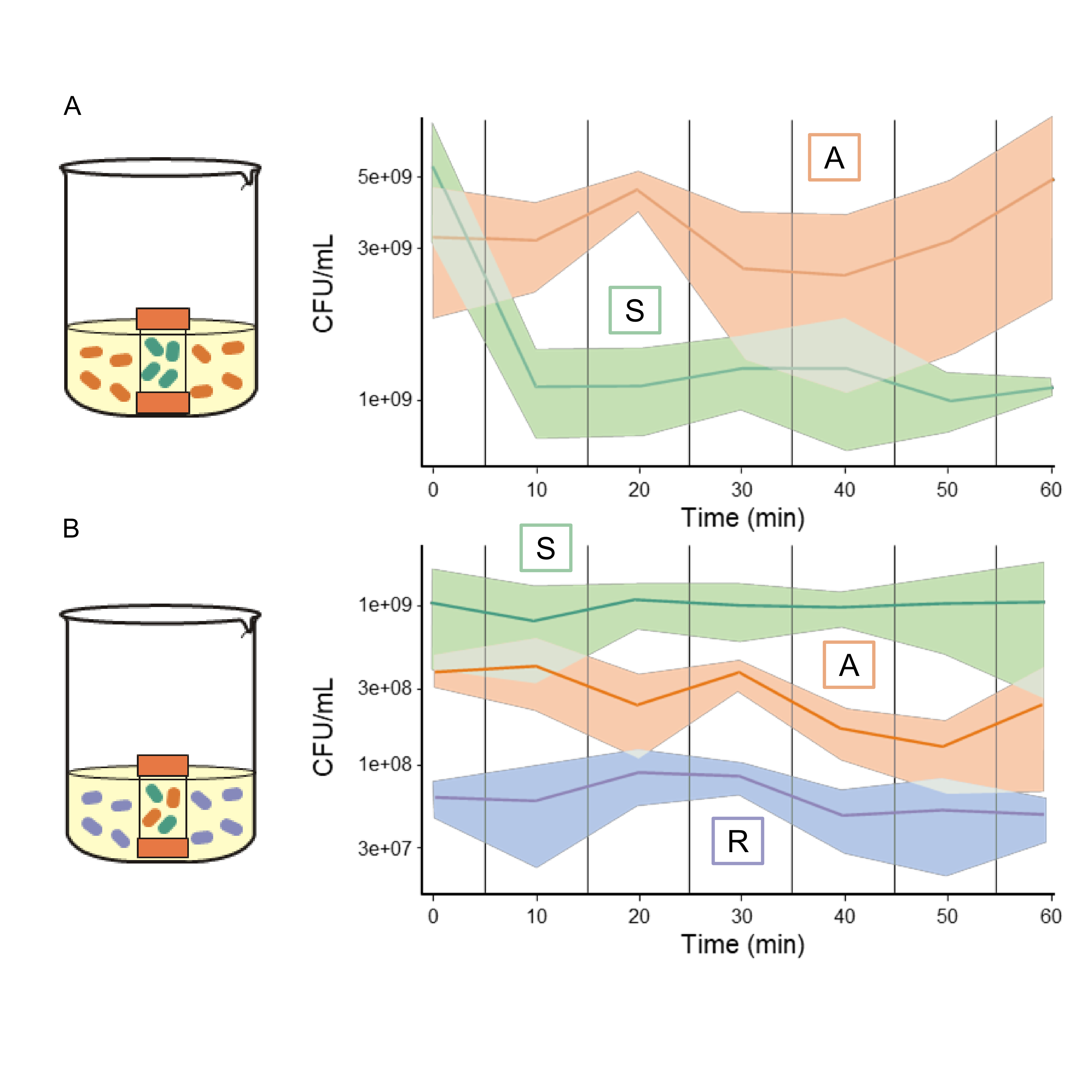

Supplement: Supplementary file 5 [file Image_4.tiff]

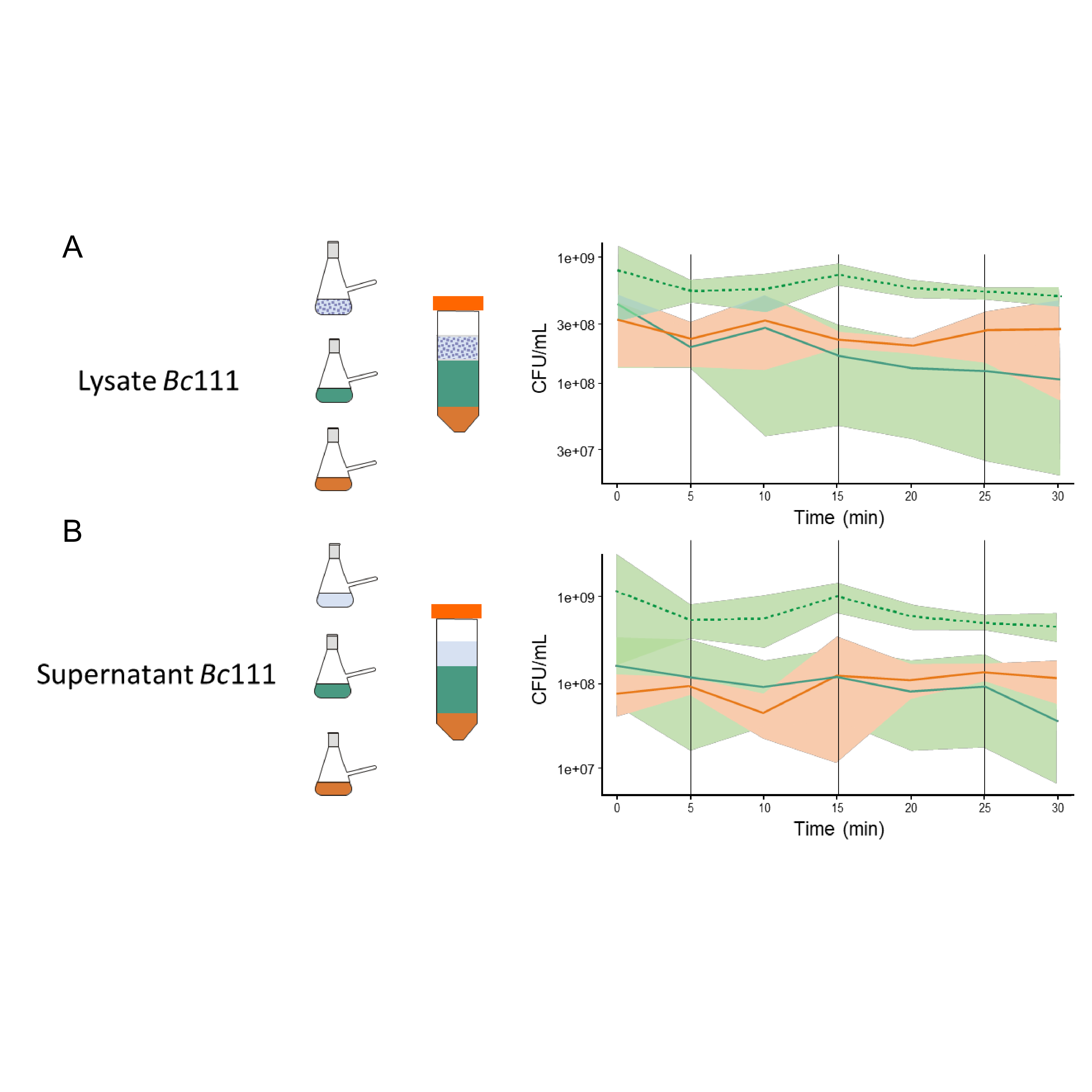

Supplement: Supplementary file 6 [file Image_5.tiff]
